# Anatomical and hormonal factors determining the development of haploid and zygotic embryos of oat (*Avena sativa* L.)

**DOI:** 10.1038/s41598-021-04522-y

**Published:** 2022-01-11

**Authors:** Kinga Dziurka, Michał Dziurka, Ewa Muszyńska, Ilona Czyczyło-Mysza, Marzena Warchoł, Katarzyna Juzoń, Kamila Laskoś, Edyta Skrzypek

**Affiliations:** 1 Department of Biotechnology, The Franciszek Górski Institute of Plant Physiology, Polish Academy of Sciences, Niezapominajek 21, 30-239 Kraków, Poland; 2 Department of Developmental Biology, The Franciszek Górski Institute of Plant Physiology, Polish Academy of Sciences, Niezapominajek 21, 30-239 Kraków, Poland; 3 Department of Botany, Institute of Biology, Warsaw University of Life Sciences-SGGW, Nowoursynowska 159, Building 37, 02-776 Warsaw, Poland

**Keywords:** Plant development, Plant embryogenesis

## Abstract

A critical step in the production of doubled haploids is a conversion of the haploid embryos into plants. Our study aimed to recognize the reasons for the low germination rate of *Avena sativa* haploid embryos obtained by distant crossing with maize. Oat cultivars of ‘Krezus’ and ‘Akt’ were investigated regarding embryo anatomy, the endogenous phytohormone profiles, and antioxidant capacity. The zygotic embryos of oat were used as a reference. It was found that twenty-one days old haploid embryos were smaller and had a less advanced structure than zygotic ones. Morphology and anatomy modifications of haploid embryos were accompanied by extremely low levels of endogenous auxins. Higher levels of cytokinins, as well as tenfold higher cytokinin to auxin ratio in haploid than in zygotic embryos, may suggest an earlier stage of development of these former. Individual gibberellins reached higher values in ‘Akt’ haploid embryos than in the respective zygotic ones, while the differences in both types of ‘Krezus’ embryos were not noticed. Additionally to the hormonal regulation of haploid embryogenesis, the poor germination of oat haploid embryos can be a result of the overproduction of reactive oxygen species, and therefore higher levels of low molecular weight antioxidants and stress hormones.

## Introduction

Doubled haploids (DHs) are widely used in breeding programs to shorten the time needed for new cereal cultivars production. The advantages of using DH lines include acceleration of the breeding process by reducing the time to obtain homozygous lines as compared with inbreeding, and increased efficiency of selection in early generations. They can be successfully used as parents for F1 generation in hybrid seed production.

In oat (*Avena sativa* L.), DHs can be produced by androgenesis in anther^1–3^ or isolated microspore cultures^4^, but an intergeneric hybridization between oat and maize (*Zea mays* L. var. *saccharata*) seems to be a more promising and efficient method^5–7^. The method involves pollination of oat with maize followed by ‘embryo rescue’, its in vitro conversion into a haploid plant, and doubling the chromosomes with colchicine.

So far, more attention has been paid to the production of DHs than to the study of the mechanisms responsible for the induction and formation of haploid embryos. In our previous research, we determined the influence of the genotype and type of auxin on the production of oat DH lines^8^. We selected the type of medium, the concentration of maltose, and the content of growth regulators to enable the conversion of haploid embryos into plants^9–11^. The effect of light, size, and age of the isolated embryo on regeneration of the haploid embryos was also assessed^10,12,13^. Nowakowska^13^ isolated embryos after 2, 3, 4, and 5 weeks and demonstrated that regeneration of 21-day-old embryos was the highest, therefore, in subsequent studies, haploid embryos were always isolated after 3 weeks. As a consequence, a detailed protocol was developed describing the technique for obtaining DHs by oat × maize crosses^14^. The molecular and hormonal mechanisms underlying the oat × maize hybridization are still poorly understood, although it is known that centromere histone H3 (CENH3) plays a significant role in uniparental chromosome elimination^15^. Manipulation of a single centromere protein allows for easy elimination of mutant chromosomes and obtaining a haploid progeny^16^.

Oat haploid embryos frequently do not produce endosperm, i.e. nutrient tissue, or the endosperm is residual. Therefore, to enable their further development, an embryo rescue procedure is necessary^17^. The embryo excised from the ovary is placed directly on a medium under sterile conditions in vitro. A critical step in DH lines production is a conversion of the haploid embryos into plants. Understanding the role of endogenous phytohormones in the germination of haploid embryos could help determine the composition of the medium used in the embryo culture. Due to the low efficiency of the method, low weight of haploid embryos, and huge labor intensity the distant crossing requires, the content of endogenous phytohormones in haploid embryos has not been studied yet. However, we know the general hormonal pattern during seed development in Arabidopsis and maize^18^. Auxins content varies during seed development. They accumulate a few or a dozen days beyond pollination and then fall during embryo maturation. A similar trend is also observed in other species, such as *Daucus carota*, *Pisum sativum,* and *Triticum aestivum*^19–21^. Cytokinins and brassinosteroids are high during cell divisions and then decrease with time. Two peaks of gibberellins (GAs) are observed at the end of the differentiation and maturation phases. Abscisic acid (ABA) acts as a GAs antagonist and its level rises when the seed enters dormancy^18^. Additionally, hormonal profiles of oat ovaries in which haploid embryos developed and ovaries in which no embryo developed were investigated, showing that the formation of a haploid embryo is accompanied by an increase in indole-3-acetic acid (IAA), trans-zeatin (t-Z), and kinetin (KN)^22^.

The average frequency of oat haploid embryos obtained by distant crossing with maize is 7.8% (7.8 embryos per 100 florets), and the regeneration rate is about 10% (10% of the embryos germinate into haploid plants)^7^. Meanwhile, for wheat, these values are respectively 11.3% and 76%^23^. Therefore the our study aimed to identify the causes of poor germination of oat haploid embryos. We did this by analyzing the anatomy, preparing endogenous phytohormone profiles, and verifying the level of antioxidants in haploid embryos vs. zygotic embryos. A combination of these methods allowed us to work out a novel research approach. According to our knowledge, this was the first study of this type. The value of the work is further increased by the methodological approach, thanks to which determination of endogenous phytohormones and antioxidants in small samples, such as haploid embryos, was possible.

## Materials and methods

### Plant materials

The study involved oat (*Avena sativa* L.) of cultivars ‘Krezus’ (hulled) and ‘Akt’ (hull-less) and maize (*Zea mays* L. var. *saccharata*), cv. ‘Waza’. The plants were placed individually in pots filled with a mixture of garden soil and sand (3:1) and grown in a greenhouse at 16 h photoperiod (photosynthetic active radiation (PAR) of 600 μmol m^−2^ s^−1^), and at 21/17 °C day/night. The authors confirm that all methods used were performed in accordance with the relevant guidelines and legislation.

### Embryo production

Oat plants intended for the production of haploid embryos were manually emasculated when half of the head was visible above the flag leaf. Then, after two days, the flowers were pollinated with maize pollen, and the next day they were treated with auxin (2,4-D) solution (100 mg dm^−3^), as previously described^7–9,12,22^. Twenty-one days after the pollination (DAP), enlarged ovaries were collected, individually opened under a microscope and any haploid embryos found were isolated. Haploid embryos were compared with zygotic ones. Oat plants intended for the production of zygotic embryos were monitored daily during their flowering stage. The day on which withered stamens could be observed was considered the first DAP. To exclude the effects of 2,4-D, used for the production of haploid embryos, on the endogenous composition of phytohormones, flowers in half of the oat plants were sprayed with auxin solution (100 mg dm^−3^) one day after the self-pollination. Twenty-one days after self-pollination the set oat kernels were collected, the embryos were separated from the endosperm and stored for further analyses. Additional oat plants cv. ‘Krezus’ were used to monitor the IAA content in the developing kernels. For this purpose, the ovaries with developing embryos were collected 1, 3, 5, 7, 9 DAP, and embryos 14 and 21 DAP were isolated. The analysis of the IAA content was performed by the HPLC method in the same way as for the other tested embryos. The results are presented in Supplementary Fig. 1.

### Microscopic sections of haploid and zygotic oat embryos

Isolated embryos were embedded separately in 1% agar droplets (low melting point). The samples were fixed in a mixture of 2.5% glutaraldehyde and 1.5% paraformaldehyde in 0.1 M sodium phosphate buffer (pH 7.0) for 2.5 h, rinsed four times in the same buffer and post-fixed in a solution of 2% osmium tetroxide/sodium-phosphate buffer for 3 h at 4 °C. Then, the samples were dehydrated through a graded ethanol series and substituted by propylene oxide before embedding in epoxy resin Epon 812 (SERVA). The resin was polymerized at 60 °C for 24 h. Semi-thin (3 µm) sections were prepared using a microtome (Jung RM 2065, Leica Microsystems), stained with methylene blue and azure A, and examined under a light microscope (Olympus-Provis, Japan).

### Extraction and quantification of phytohormones and related compounds

Extraction and quantification of phytohormones and related compounds were performed according to^22^. The isolated embryos (haploid and zygotic) were lyophilized, weighted, and pulverized in a mixing mill (MM 400, Reatch, Kroll, Germany) with zirconium oxide beads. The samples spiked with stable isotope-labeled internal standards were extracted to methanol/water/formic acid mixture (MeOH/H_2_O/HCOOH 15/4/1 v/v/v)^24^ with homogenizing beads not removed. The extraction was repeated twice and combined extracts were evaporated under N_2_, then resuspended in 5% MeOH in 1 M HCOOH and cleaned up on SPE cartridges (BondElutPlexa PCX, 30 mg, 1 mm, Agilent, USA). The fraction containing the hormones and related compounds was evaporated under N_2_, reconstituted in 70 µl of ACN, filtered (0.22 µm nylon membrane), and used for UHPLC analyses. The system consisted of UHPLC (Agilent Infinity 1260, Agilent, Germany) and a triple quadrupole mass spectrometer (Agilent 6410, Agilent, USA) with electrospray ionization (ESI). Separation was achieved on AscentisExpres RP-Amide analytical column (2.7 μm, 2.1 mm × 150 mm; Supelco, USA) at a linear gradient of water vs. ACN both with 0.01% HCOOH. The monitored hormones included: indolebutyric acid (IBA), indole-3-acetic acid methyl ester (MeIAA), [^2^H_5_]indole-3-acetic acid methyl ester (MeIAA-D5, used as ISTD), indole-3-acetic acid (IAA), [^2^H_5_]indole-3-acetic acid (IAA-D5, used as ISTD), indole-3-carboxilic acid (I3CA), indole-3-acetyl-glutamic acid (IAA-Glu), indole-3-acetyl-aspartic acid (IAA-Asp), oxoindole-3-acetic acid (OxIAA), kinetin (K) and [^15^N_4_]kinetin (K-N15, ISTD), kinetin-9-riboside (KR), N6-isopentenyladenosine (IPD), N6-izopentenyladenine (IP), dihydrozeatin (DH-Z), *cis*-zeatin (c-Z),* trans*-zeatin (t-Z), [^15^H-N_4_]dihydrozeatin (DHZ-N15, used as ISTD), dihydrozeatin riboside (DH-Z-R), *cis*-zeatin-9-riboside (c-Z-R), *trans*-zeatin-9-riboside (t-Z-R), [^2^H_5_]*trans*-zeatin riboside (t-ZR-D5, used as ISTD),* cis*-zeatin-O-glucoside (c-Z-O-G), *cis*-zeatin-7-glucoside (c-Z-7-G), *trans*-zeatin-O-glucoside (t-Z-O-G), *trans*-zeatin-7-glucoside (t-Z-7-G), gibberellin A_9_ (GA9), gibberellin A_7_ (GA7), gibberellin A_4_ (GA4), gibberellin A_5_ (GA5), gibberellin A_6_ (GA6), gibberellin A_1_ (GA1), gibberellic acid (GA3), [^2^H_2_]gibberellic acid (GA3-D2, used as ISTD), gibberellin A_8_ (GA8), salicylic acid (SA), [^2^H_4_]salicylic acid (SA-D4, used as ISTD), 12-oxo-phytodenoic acid (12-oxo-PDA), jasmonic acid (JA), jasmonic acid methyl ester (MeJA), *cis,trans*-abscisic acid (ABA), [^2^H_6_]*cis,trans*-abscisic acid (ABA-D6, used as ISTD), abscisic acid glucosyl ester (ABA-glc), *trans,trans*-abscisic acid (tt-ABA). Multiple reactions monitoring (MRM) transitions were used for the identification and quantization of all compounds of interest (Table S1). All standards were from OlChemim (Olomounc, Czech Republic) at the highest available purity, whereas all solvents were of HPLC grade from Sigma-Aldrich (Poznań, Poland).

### Antioxidant activity

The content of non-enzymatic, low-molecular-weight antioxidants was determined using the CUPRAC (cupric ion reducing antioxidant capacity) method^25^ with some modifications^26^. Twenty microliter aliquots of methanolic extract acquired in the phytohormone extraction were transferred to 96-well plates containing 50 μl of 10 mM Cu^2+^ aqueous solution, 50 μl 7.5 mM neocuproin in methanol and 80 μl 1 M ammonium acetate buffer (pH = 7) in each well. The plates were incubated for 5 min at 25 °C and their absorbance was read at 450 nm (Synergy II, Biotek, USA, software: Gen5, Biotek, USA). The content of antioxidants was expressed as nmol/mg Trolox equivalents.

### Statistical analysis

The means and standard errors (SE) were calculated for three replicates. One-way analysis of variance (ANOVA) was used to determine the effect of the embryo type on their phytohormone and antioxidant profiles within each cultivar. Duncan’s multiple range test was used to separate the means at the *P* < 0.05 probability level. All the data were analyzed with STATISTICA 10.0 (Stat-Soft, Inc., USA) software package.

## Results

### The morphology and anatomy of oat’s embryos

**Figure 1:**
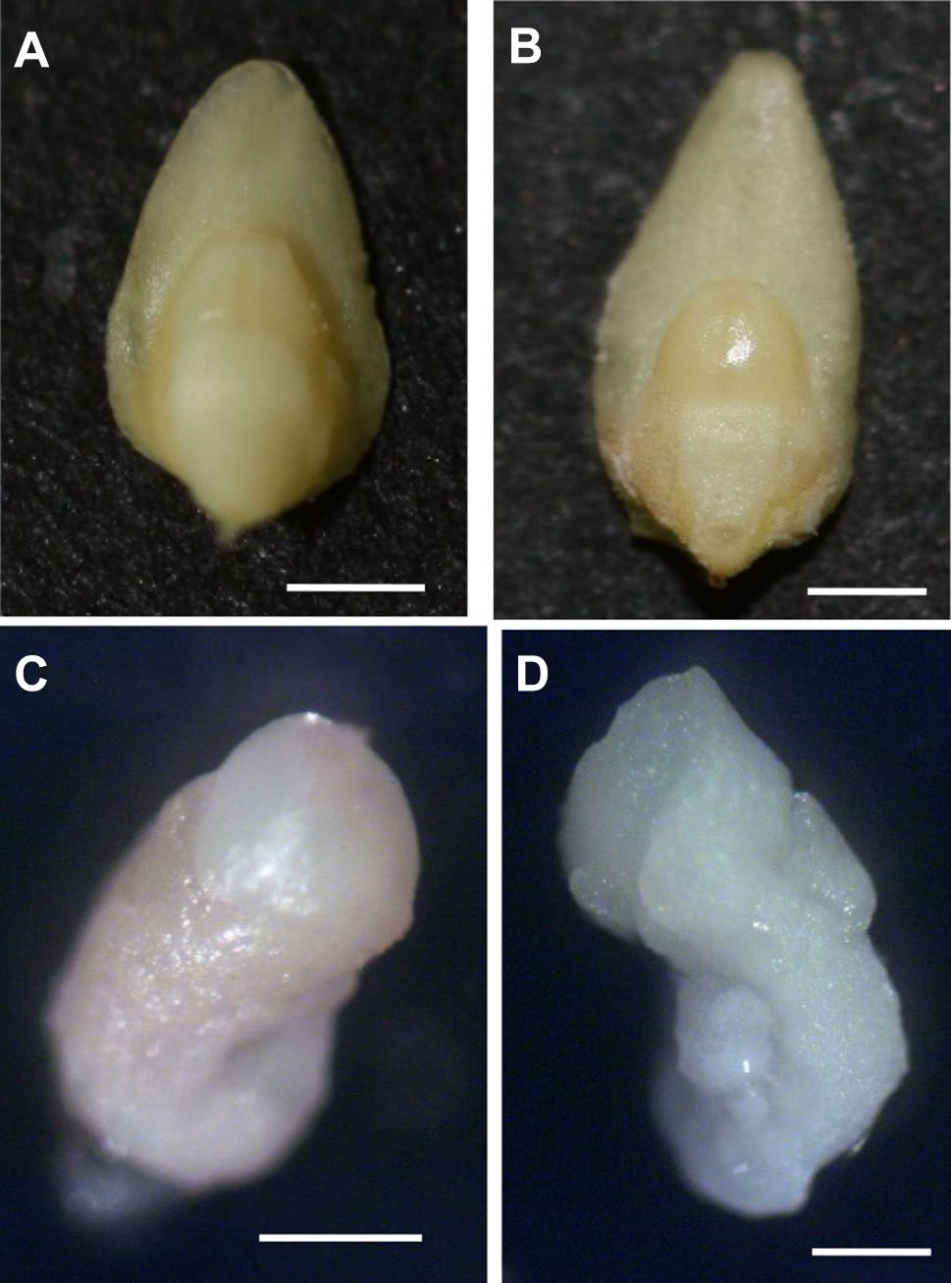
Morphology of oat embryos. (**A**) zygotic embryo cv. ‘Krezus’, (**B**) zygotic embryo cv. ‘Akt’, (**C**) haploid embryo cv. ‘Krezus’, (**D**) haploid embryo cv. ‘Akt’. Scale: (**A**,**B**) 1000 µm, (**C**,**D**) 500 µm.

The 21-day old zygotic embryos differed in their morphology from the haploid embryos (Fig. 1). The first ones (Fig. 1A,B) were clearly bipolar, about 4 mm long, cream-colored. They had properly developed scutellum with smooth edges. The haploid embryos (Fig. 1C,D) were smaller (ca. 1.5 mm), white, and of irregular shape. Their shoot and root apical meristem parts were often difficult to identify.

**Figure 2:**
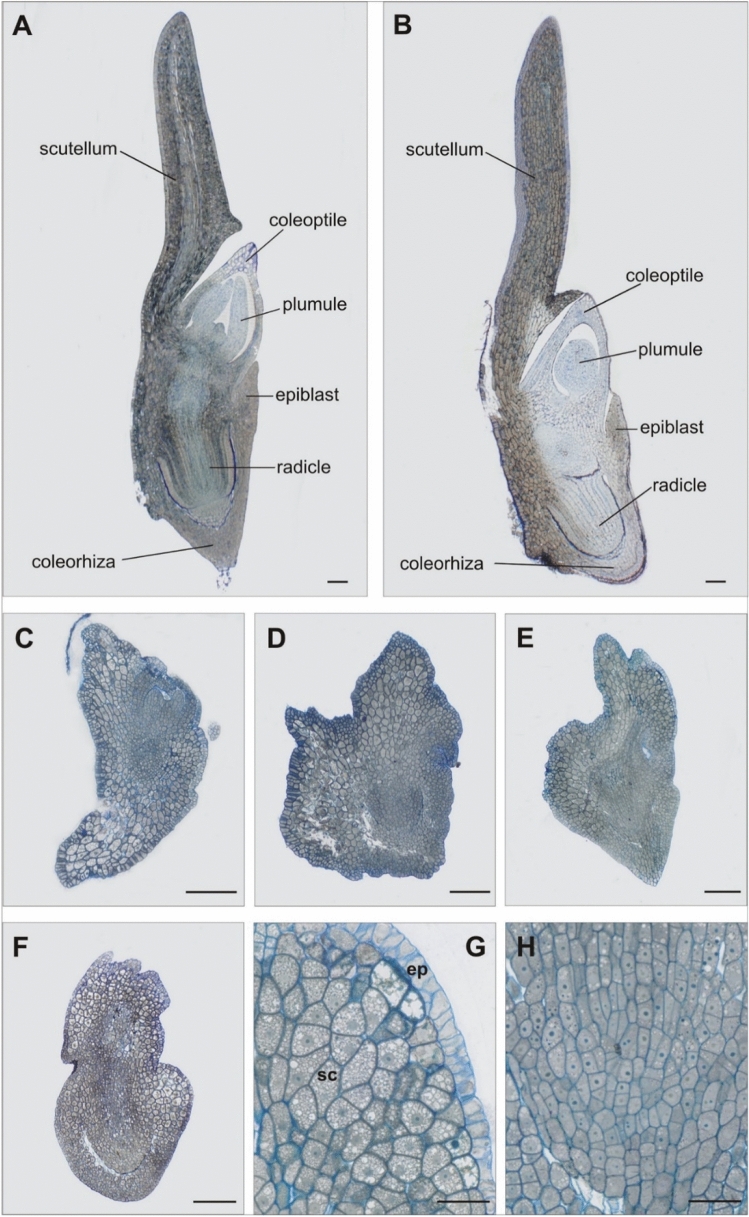
Anatomical sections of oat embryos. (**A**) zygotic embryo cv. ‘Krezus’, (**B**) zygotic embryo cv. ‘Akt’, (**C**,**D**) haploid embryos cv. ‘Krezus’, (**E**–**H**) haploid embryos cv. ‘Akt’, (**G**) greater magnification of vacuolized large parenchyma cells building the scutellum (sc) and epithelial layer (ep), (**H**) division zone of the apical part of the root. Scale: (**A**–**F**) 200 µm; (**G**,**H**) 50 µm.

Microscopic analysis of longitudinal sections revealed further differences between the embryos (Fig. 2). The zygotic embryos (Fig. 2A,B) had a repetitive and regular structure, with a fully formed shoot apical meristem surrounded by a coleoptile, and a root apical meristem with forming lateral roots surrounded by a coleorhiza. Their scutellum with clearly visible epithelial cells and the epiblast were properly developed. The haploid embryos (Fig. 2C–H) were more diverse, with less ordered and less advanced structure. They differed in shape, especially of the scutellum (Fig. 2C–H), which was surrounded from the outside with a layer of elongated cells (probably of epithelial nature), and had its interior filled with thin-walled, highly vacuolated parenchymal cells (Fig. 2G). The other parts of the embryos were made of small, meristematic cells filled with dense cytoplasm that formed zones of intense cell divisions denoting the root and/or stem areas of the haploid embryo (Fig. 2H).

### Endogenous profile of phytohormones in oat haploid embryos

**Figure 3:**
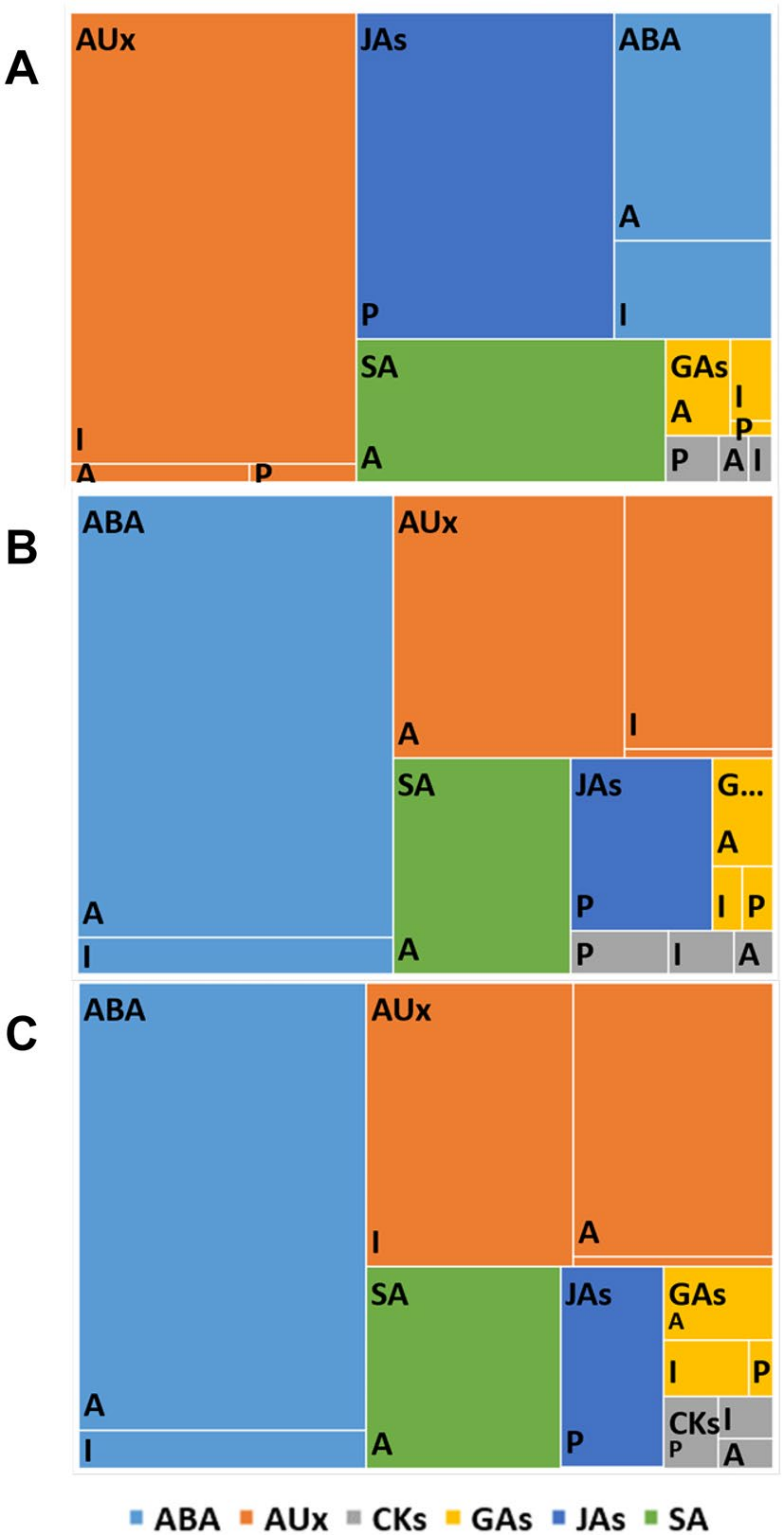
A treemap chart of the hormonal profile of oat cv. ‘Krezus’ embryos. Panel (**A**) haploid embryos, Panel (**B**) zygotic embryos, Panel (**C**) zygotic embryos from ovaries treated with 2,4-D. The total area represents the amount of all measured hormones. Rectangles of different colors: *AUx* auxins (orange), *JAs* jasmonates (navy blue), *ABA* abscisic acid (blue), *GAs* gibberellins (yellow), *SA* salicylic acid (green), and *CKs* cytokinins (grey) represent the share of each compound group divided in to active (A), inactivated (I), and precursor (P) fractions.

**Figure 4:**
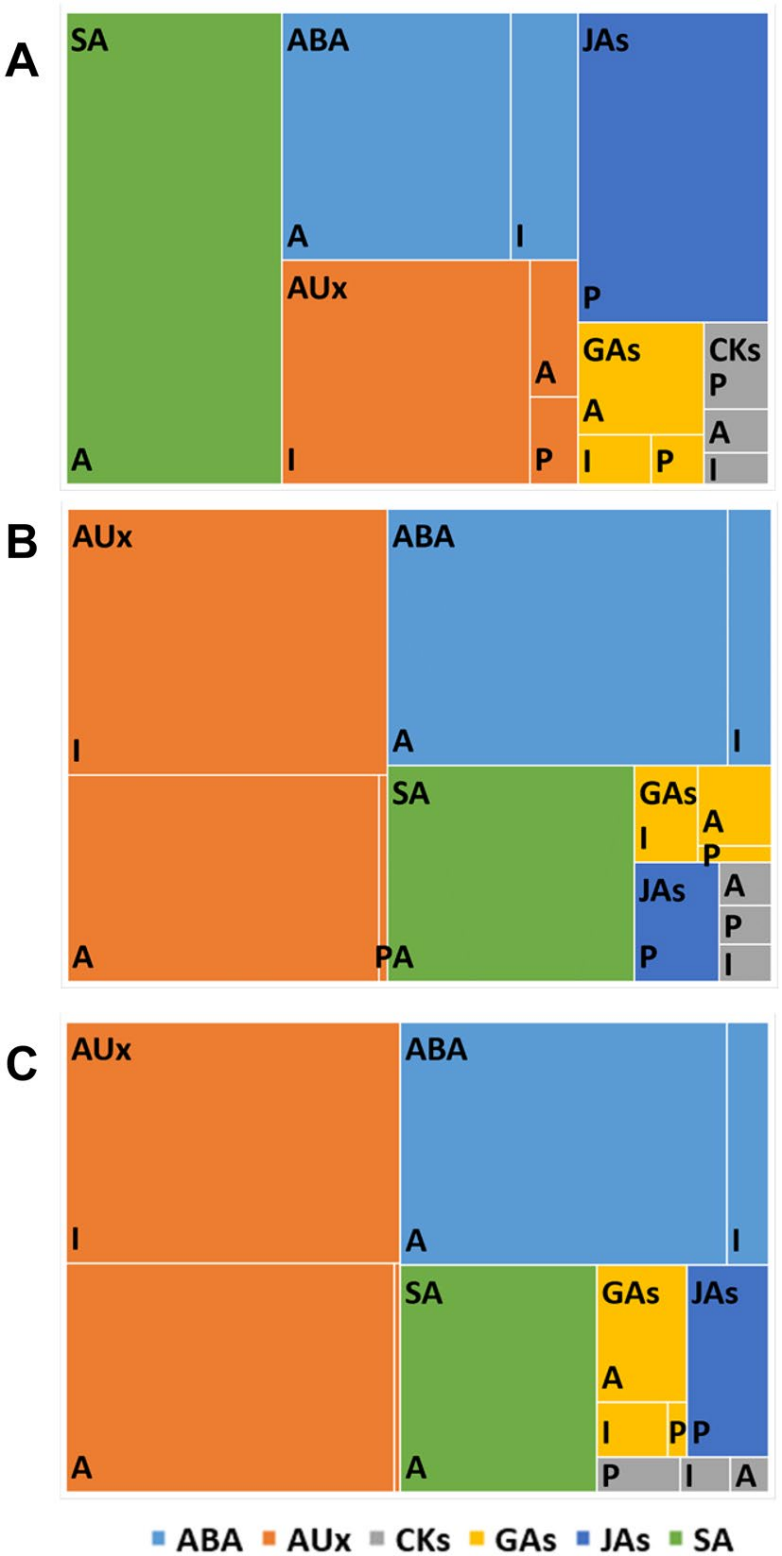
A treemap chart of the hormonal profile of oat cv. ‘Akt’ embryos. Panel (**A**) haploid embryos, Panel (**B**) zygotic embryos, Panel (**C**) zygotic embryos from ovaries treated with 2,4-D. The total area represents the amount of all measured hormones. Rectangles of different colors: *AUx* auxins (orange), *JAs* jasmonates (navy blue), *ABA* abscisic acid (blue), *GAs* gibberellins (yellow), *SA* salicylic acid (green), and *CKs* cytokinins (grey) represent the share of each compound group divided in to active (A), inactivated (I), and precursor (P) fractions.

Distant crossing of oat and maize aimed at obtaining haploid plants is based on 2,4-D used for treating oat ovaries after pollination with maize pollen. One may wonder if 2,4-D alters the endogenous hormonal profile of the embryos. As previously described since it is impossible to obtain haploid embryos without treating oat ovaries with the synthetic auxin, our experiments involved zygotic embryos. To exclude the effects of 2,4-D on the endogenous composition of phytohormones of embryos we determined the content of auxins, cytokinins, gibberellins, and stress-related hormones in zygotic embryos and in zygotic embryos isolated from ovaries treated with 2,4-D. Analysis of hierarchical tree diagrams for the zygotic (Figs. 3B and 4B) and zygotic embryos whose ovaries were treated with 2,4-D (Figs. 3C and 4C) showed only slight changes in the proportion of active and inactivated forms of auxins in both cultivars.

These differences were not statistically significant (Tables 1, 2). A similar pattern as for auxins was observed for cytokinins (except for inactivated form c-Z-O-G in cv. ‘Krezus’), gibberellins, and stress hormones. In the zygotic embryos of cv. ‘Krezus’ the most abundant hormone was ABA and related compounds (Fig. 3B), while the embryos of cv. ‘Akt’ were the richest in auxins (Fig. 4B). The zygotic embryos of cv. 'Akt' contained also more gibberellins than those of cv. 'Krezus' (Tables 1, 2).

**Table 1:** Hormonal profile of cv. ‘Krezus’ embryos.

Compound [pmol/g] DW	Function	Haploid embryo	Zygotic embryo	Zygotic embryo after 2,4-D treatment
**Auxins**				
IBA	Precursor	298.7 ± 37.2 a	66.9 ± 6.0 b	83.5 ± 11.0 b
IAA	Active	502 ± 10.3 b	3059.8 ± 369.0 a	2247.8 ± 648.1 a
13CA	Inactivated	18,501.3 ± 2164.3 a	294.6 ± 12.9 b	714.8 ± 287.5 b
MeIAA	Inactivated	481.4 ± 83.1 a	167.5 ± 38.1 a	351.5 ± 166.1 a
IAA-Glu	Inactivated	695.2 ± 69.5 a	346.0 ± 14.1 b	306.0 ± 11.5 b
IAA-Asp	Inactivated	616 ± 29.4 a	1007.6 ± 208.2 a	918.2 ± 228.7 a
OxIAA	Inactivated	232.7 ± 77.1 a	81.5 ± 34.1 a	127.7 ± 64.7 a
Sum of inactivated forms		20,526.6	1897.2	2418.2
**Cytokinins**				
K		nd	nd	nd
K-R		nd	nd	nd
IPD	Precursor	3.6 ± 0.7 a	2.0 ± 0.4 a	2.4 ± 0.3 a
IP	Precursor	320.2 ± 29.3 a	178.1 ± 13.6 b	138.0 ± 14.5 b
Sum of precursors		323.8	180.1	140.4
DH-Z	Active	102.6 ± 31.6 a	28.9 ± 10.3 b	14.0 ± 7.0 b
c-Z	Active	18.3 ± 5.5 a	38.3 ± 10.0 b	32.6 ± 7.1 a
t-Z	Active	98.4 ± 24.7 a	16.5 ± 3.5 b	20.0 ± 2.8 b
DH-Z-R	Active	8.8 ± 5.6	nd	nd
c-Z-R	Active	54.4 ± 5.4 a	23.7 ± 3.5 b	18.8 ± 8.2 b
t-Z-R	Active	4.6 ± 2.3 a	5.0 ± 0.5 a	2.4 ± 0.5 a
Sum of active forms		287.1	112.4	87.8
c-Z-O–G	Inactivated	8.9 ± 4.8 b	86.4 ± 9.3 a	38.7 ± 12.7 b
c-Z-7-G	Inactivated	31.5 ± 3.0 a	7.6 ± 0.6 b	17.3 ± 9.9 ab
t-Z-O–G	Inactivated	114 ± 13.9 a	42.2 ± 1.5 b	32.3 ± 3.9 b
t-Z-7-G	Inactivated	19.3 ± 1.2 a	4.3 ± 2.2 b	6.4 ± 1.1 b
Sum of inactivated forms		173.7	140.5	94.7
**Gibberellins**				
GA9	Precursor	54.1 ± 54.1 a	79.8 ± 12.4 a	52.8 ± 4.8 a
GA5	Precursor	43.1 ± 22.6 a	18.2 ± 9.3 a	3.0 ± 3.0 a
Sum of precursors		97.2	98	55.8
GA7	Active	nd	nd	nd
GA4	Active	nd	6.4 ± 3.7 a	4.3 ± 4.3 a
GA6	Active	214.0 ± 96.0 a	96.4 ± 28.0 a	114.1 ± 36.5 a
GA1	Active	61.7 ± 31.0 a	30.4 ± 15.9 a	nd
GA3	Active	707.6 ± 328.8 a	195.5 ± 85.0 a	207.4 ± 70.0 a
Sum of active forms		983.3	328.8	325.7
GA8	Inactivated	544.8 ± 379.8 a	98.2 ± 24.9 a	196.3 ± 26.5 a

Values are means of three replicates ± standard error. Means indicated by the same letter do not significantly differ at *P* < 0.05 according to one-way ANOVA and post hoc Duncan’s test.

**Table 2:** Hormonal profile of cv. ‘Akt’ embryos.

Compound [pmol/g] DW	Function	Haploid embryo	Zygotic embryo	Zygotic embryo after 2,4-D treatment
**Auxins**				
IBA	Precursor	517.8 ± 111.5 a	51.0 ± 12.0 b	50.9 ± 23.1 b
IAA	Active	807.3 ± 23.3 a	1814.4 ± 690.6 a	2718.5 ± 1150.6 a
13CA	Inactivated	nd	705.8 ± 30.7 a	720.5 ± 28.1 a
MeIAA	Inactivated	916.6 ± 213.2 a	186.0 ± 82.0 b	317.5 ± 107.0 b
IAA-Glu	Inactivated	1302.3 ± 178.5 a	255.9 ± 65.3 b	259.9 ± 36.1 b
IAA-Asp	Inactivated	3052.1 ± 991.4 a	1092.1 ± 347.4 a	1314.0 ± 122.6 a
OxIAA	Inactivated	299.7 ± 32.8 a	169.7 ± 15.8 a	316.0 ± 132.9 a
Sum of inactivated forms		5570.7	2409.5	2927.9
**Cytokinins**				
K		nd	nd	nd
K-R		nd	nd	nd
IPD	Precursor	1.2 ± 1.04 a	0.7 ± 0.5 a	1.0 ± 0.2 a
IP	Precursor	586.9 ± 97.4 a	43.7 ± 22.0 b	92.8 ± 15.7 b
Sum of precursors		588.1	44.4	93.8
DH-Z	Active	153.4 ± 66.6 a	10.2 ± 8.2 b	6.8 ± 4.5 b
c-Z	Active	61.4 ± 3.6 a	26.4 ± 1.3 b	24.1 ± 6.0 b
t-Z	Active	130.8 ± 52.0 a	26.2 ± 6.7 ab	18.1 ± 4.6 b
DH-Z-R	Active	nd	nd	nd
c-Z-R	Active	70.2 ± 2.5 a	9.9 ± 1.8 b	9.0 ± 2.0 b
t-Z-R	Active	25.1 ± 0.3 a	2.9 ± 0.4 b	3.1 ± 0.3 b
Sum of active forms		287.1	75.6	61.1
c-Z-O–G	Inactivated	nd	17.1 ± 12.2 a	26.6 ± 5.7 a
c-Z-7-G	Inactivated	31.3 ± 15.6 a	6.0 ± 1.7 a	5.7 ± 0.8 a
t-Z-O–G	Inactivated	185.2 ± 15.4 a	28.2 ± 11.3 b	26.2 ± 3.6 b
t-Z-7-G	Inactivated	33.0 ± 6.6 a	3.0 ± 1.6 b	6.3 ± 1.4 b
Sum of inactivated forms		249.5	54.3	64.8
**Gibberellins**				
GA9	Precursor	314.7 ± 49.4 a	35.1 ± 21.7 b	39.6 ± 4.0 b
GA5	Precursor	7.9 ± 7.9	nd	nd
Sum of precursors		322.7	35.1	39.6
GA7	Active	nd	nd	40.8 ± 22.6
GA4	Active	113.4 ± 113.4 a	nd	108.9 ± 60.8 a
GA6	Active	477.4 ± 67.8 a	85.0 ± 37.0 b	101.5 ± 32.3 b
GA1	Active	nd	nd	26.7 ± 16.8
GA3	Active	1157.5 ± 221.8 a	nd	169.8 ± 96.2b
Sum of active forms		2192.5	258.2	588.1
GA8	Inactivated	444.1 ± 340.0 a	173.2 ± 89.4 a	140.4 ± 84.9a

Values are means of three replicates ± standard error. Means indicated by the same letter do not significantly differ at *P* < 0.05 according to one-way ANOVA and post hoc Duncan’s test.

The haploid embryos of both cultivars had a higher level of IBA precursor than the zygotic embryos (Tables 1, 2). Interestingly, in both cultivars, the sum of inactivated auxins was greater in the haploid than in the zygotic embryos. The haploid embryos of cv. ‘Krezus’ accumulated more inactivated forms of I3CA and IAA-Glu (18,501.3 and 659.2 pmol/g DW, respectively) and lower levels of IAA (502.0 pmol/g DW ) than the zygotic embryos (294.6; 346.0 and 3059.8 pmol/g DW, respectively) (Table 1). In the zygotic embryos of cv. ‘Krezus’ the active forms of auxins were close twice as abundant as the inactivated ones, while in the haploid embryos this relationship was reversed. The haploid embryos contained several times more inactivated than active auxin forms. These relationships are clearly depicted in the hierarchical tree diagram (Fig. 3A,B). In the haploid embryos of cv. ‘Akt’ we detected a few times greater amounts of inactivated forms of auxins MeIAA and IAA-Glu than in the zygotic embryos (Table 2). At the same time, similarly to cv. ‘Krezus’, we confirmed insignificantly lower content of IAA (807.3 pmol/g DW) compared to zygotic embryos (1814.4 pmol/g DW). Interestingly, the haploid embryos of cv. ‘Akt’ did not contain I3CA that was accumulated to a very high degree in the haploid embryos of cv. ‘Krezus’.

The haploid embryos of cv. ‘Krezus’ contained about 1.5 times more cytokinin precursor IP and 2.5 times more active cytokinins than the zygotic embryos (Table 1). They were also richer in inactivated cytokinins. IPD, c-Z, and t-Z-R occurred at a similar level in both haploid and zygotic embryos. c-Z-O-G was the only form of cytokinin more abundant in the zygotic (86.4 pmol/g DW) than in the haploid (8.9 pmol/g DW) embryos. In the haploid embryos of cv. ‘Akt’ we found significantly greater differences in the content of IPA cytokinin precursor, active and inactivated cytokinins (by 13; 4  and 5 times, respectively) than in zygotic ones. Similarly as for cv. ‘Krezus’, we detected no differences in IPD and t-Z content, while c-Z-O-G was more abundant in the zygotic (17.1 pmol/g DW) than the haploid embryos where it was below the detection limit.

The haploid and zygotic embryos of cv. ‘Krezus’ did not differ significantly in their gibberellin content, while in cv. ‘Akt’ the haploid embryos contained several times more precursor GA9 and gibberellin A6 than the zygotic ones. GA4, GA5, GA3 were also present in the haploid embryos but were not detected in the zygotic ones.

SA, JA, ABA, and their derivatives have been grouped . They are called stress-related hormones and are presented in Tables 3 and 4. The haploid embryos of cv. ‘Krezus’ accumulated more stress hormones, i.e. SA, 12-oxo-PDA and JA , than the zygotic embryos (Table 3).

**Table 3:** Stress-related hormones in cv. ‘Krezus’ embryos.

Compound [pmol/g] DW	Function	Haploid embryo	Zygotic embryo	Zygotic embryo after 2,4-D treatment
SA	Active	7033.4 ± 414.8 a	1937.0 ± 581.0 b	1608.2 ± 424.9 b
12-oxo-PDA	JA precursor	4647.8 ± 627.9 a	448.6 ± 136.3 b	415.4 ± 191.9 b
JA	JA-Ile precursor	8742.6 ± 1394.4 a	784.0 ± 75.9 b	429.2 ± 83.9 b
MeJA	Inactivated	8.7 ± 4.7 a	nd	5.3 ± 5.3 a
ABA	Active	4772.7 ± 232.1 a	6226.4 ± 1251.4 a	4870.1 ± 1002.4 a
ABA-glc	Inactivated	2470.5 ± 53.5 a	573.2 ± 86.8 b	450.2 ± 61.0 b
tt-ABA	Inactivated	956.5 ± 294.9 a	806.8 ± 442.5 a	416.6 ± 50.6 a

Values are means of three replicates ± standard error. Means indicated by the same letter do not significantly differ at *P* < 0.05 according to one-way ANOVA and post hoc Duncan’s test.

**Table 4:** Stress-related hormones in cv. ‘Akt’ embryos.

Compound [pmol/g] DW	Function	Haploid embryo	Zygotic embryo	Zygotic embryo after 2,4-D treatment
SA	Active	12,593.8 ± 2454.7 a	1503.6 ± 339.6 b	1615.4 ± 227.6 b
12-oxo-PDA	JA precursor	3507.1 ± 471.0 a	85.9 ± 2.0 b	229.5 ± 49.4 b
JA	JA-Ile precursor	3782.0 ± 469.1 a	198.3 ± 42.0 b	335.4 ± 65.5 b
MeJA	Inactivated	nd	nd	nd
ABA	Active	5979.7 ± 330.0 a	2204.5 ± 607.8 b	2488.6 ± 66.0 b
ABA-glc	Inactivated	2058.7 ± 237.4 a	314.9 ± 79.3 b	367.3 ± 19.3 b
tt-ABA	Inactivated	1020.4 ± 98.3 a	260.2 ± 135.6 b	388.6 ± 187.1 b

Values are means of three replicates ± standard error. Means indicated by the same letter do not significantly differ at *P* < 0.05 according to one-way ANOVA and post hoc Duncan’s test.

The level of ABA was similar in both types of embryos but the inactivated form ABA-glc (2470.5 pmol/g DW) was four times more abundant in the haploid embryos than in the zygotic ones. In turn, the haploid embryos of cv. ‘Akt’ contained more of all stress hormones and their precursors and inactivated forms than the zygotic embryos (Table 4). While the zygotic embryos contained four times more active ABA (2204.5 pmol/g DW) than sum of inactivated forms (ABA-glc, tt-ABA), in haploid embryos this difference was considerably smaller.

### Antioxidant system of oat haploid embryos

**Figure 5:**
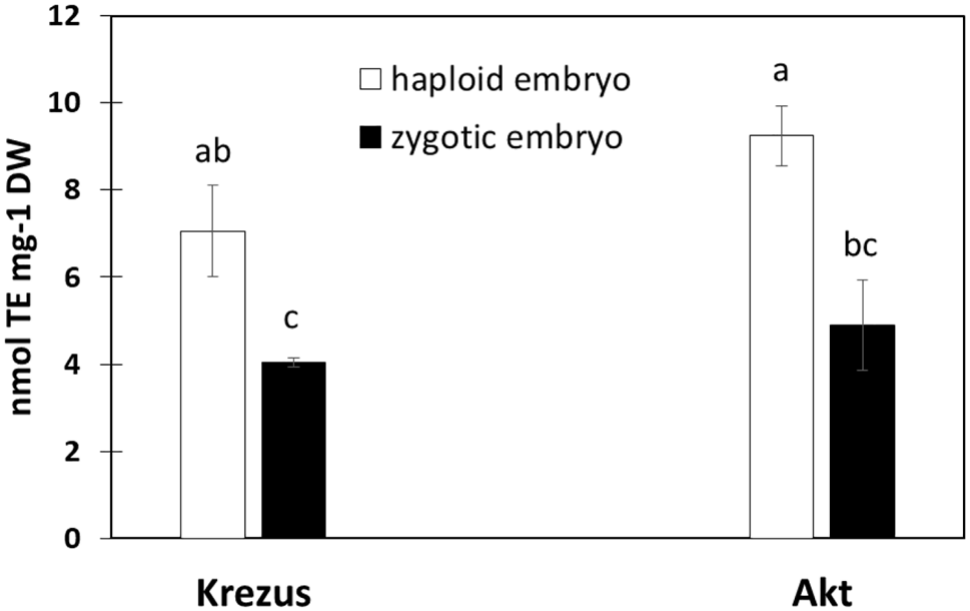
Total antioxidants measured by CUPRAC assay in haploid and zygotic embryos of oat cvs. ‘Krezus’ and ‘Akt’. Values expressed as Trolox equivalents. Different letters indicate statistically significant differences at *P* < 0.05 according to two-way ANOVA and post hoc Duncan’s test.

Significant amounts of stress-related hormones (SA, ABA, JA, and their related compounds) measured in haploid embryos have raised the question of whether oxidative stress is present in haploid embryos. Therefore, we measured the level of antioxidants in the tested embryos. We evaluated the level of non-enzymatic low molecular weight antioxidants with the CUPRAC method and revealed their twice as high concentration in the haploid than in the zygotic embryos (Fig. 5).

## Discussion

Haploid oat embryos, although important in the process of obtaining DHs, are not a popular research object. Understanding the causes of poor germination of haploid embryos can significantly improve the efficiency of DH lines production and accelerate breeding work. Our research is unique in that it provides an interdisciplinary approach to combine morphological and anatomical features with changes in the composition of endogenous phytohormones involved in the morphogenesis process.

Despite the same age (21 DAP), oat haploid embryos were smaller and had a less advanced anatomical structure than the zygotic embryos. Similarly, wheat zygotic embryos were 61% wider and by 48% longer than the haploid embryos resulting from wheat with maize cross^27^. The low percentage of germinating haploid oat embryos per 100 emasculated florets^8,10^ can be explained by the multidimensional immaturity of embryos, which was demonstrated in the presented research. Additionally, the formation of the haploid embryos is not accompanied by the development of endosperm that is considered an integrator of the various compartments and genetic programs involved in seed development^28^, and this may also negatively affect the embryo condition. Dark coloration of the embryonic disc in the zygotic embryos indicated a stronger binding of osmium. Osmium is a lipophilic element, so it can be concluded that the parenchymal cells of the scutellum contain large amounts of fat. This observation is consistent with the information that each parenchymal cells contain several protein bodies suspended in a droplet of fat^29^. In haploid embryos, the differences in preparation staining were considerably smaller, probably because these embryos did not contain as many fats as the zygotic ones. The cells of haploid embryos were strongly vacuolated, especially within the scutellum of haploid embryos cv. ‘Akt’. Their presence may be one of the signs of the incomplete structural and functional maturity of cells. Ozkara and Savaskan^27^ reported on a large number of vacuoles in wheat haploid embryos obtained by wheat and maize cross. It is believed that strong cell vacuolation may occur during developmental transitions such as embryogenesis. However, this is temporary and has to do with the replacement of lytic vacuoles by protein storage vacuoles^30^.

Data on the basic chemical composition of zygotic oat embryos can only be found in a paper by Barnes^31^, who reported on their content of nutrients, such as total acyl lipids, proteins, the composition of amino acids, fatty acids, tocopherol, and free sugars, as well as thiamine and riboflavin content in two British cultivars ‘Peniarth’ and ‘Maris Oberon’. Percival and Bandurski determined the IAA content of dehusked oat kernels, which is 8 mg/kg, of which 5.5% is free IAA and 94.5% is esterified to glucoproteins^32^. According to our knowledge, there is no information in the literature on the content of endogenous phytohormones in both haploid and zygotic oat embryos. The role of phytohormones during seed development is studied mainly in *Arabidopsis thaliana*, although there have also been studies in maize^33^, wheat^21^, oilseed rape^34^, and legumes^35,36^. The protocol for the distant crossing of oat and maize aimed at obtaining haploid plants is based on 2,4-D used for treating oat ovaries after pollination with maize pollen. To exclude the effects of 2,4-D on the endogenous composition of phytohormones of embryos we determined the content of auxins, cytokinins, gibberellins, and stress-related hormones in zygotic embryos and in zygotic embryos isolated from ovaries treated with 2,4-D. Although IAA content was slightly higher in the embryos isolated from the seeds treated with 2,4-D than in the control ones, the differences were not significant. There were also no differences in the content of the other analyzed phytohormones. Based on this experiment, it can be concluded that 2,4-D does not significantly alter the endogenous hormonal composition of haploid oat embryos. Similarly, Ribnicky et al.^37^ showed that exogenous 2,4-D had a minor effect on endogenous IAA content in carrot hypocotyl cultures, even though it promoted callus proliferation. This suggests that 2,4-D acts directly as the auxin in tissue, without altering the IAA synthesis pathway. As we found no significant differences in the content of the investigated phytohormones in control oat zygotic embryos and zygotic embryos from the ovaries treated with 2,4-D, we can assume the exposure of the ovaries to 2,4-D does not significantly affect the endogenous hormonal profile of the embryos. Therefore, the observed differences between the zygotic and haploid embryos are not due to ovary treatment with 2,4-D but to different developmental paths.

The hormonal profiles of cv. ‘Krezus’ and cv. ‘Akt’ embryos were slightly different, which was probably due to the uneven course of their maturation. The zygotic embryos of cv. ‘Krezus’ featured the highest percentage of ABA possibly indicating their dormancy. The zygotic embryos of cv. ‘Akt’ were probably less mature, as evidenced by lower accumulation of ABA and higher content of cytokinins. In wheat, the highest concentration of IAA in grain was found on 18th DAP^21^. In maize, auxin accumulation occurred between 9–11 DAP and probably initiated the kernel filling phase, with a peak occurring on 20th DAP associated with intensive embryo growth and accumulation of IAA conjugates^38^. Based on these data and sections of the zygotic embryos it may be concluded that 21 days old oat embryos are past their IAA spike. This is also confirmed by our analysis of IAA content in developing oat kernels (Supplementary Fig. 1). As the haploid embryos showed less advanced anatomy and were still developing their internal structures, one might expect higher concentrations of auxins. Meanwhile, 21 days old haploid embryos contained six and two times (cv. ‘Krezus’ and cv. ‘Akt’, respectively) less IAA than the zygotic embryos and showed higher content of IBA precursor. Auxins play a pivotal role in establishing the apical-basal axis and coordinating embryonic pattern formation^39–42^. Auxin polar transport inhibitors were shown to block the transition from the globular to the heart-shaped stage of somatic embryos in carrot^43^. Contrary to that, proper distribution of auxins is responsible for the shift from radial symmetry to bilateral polarity during embryogenesis in wheat^44^. As our oat haploid embryos were still at the organogenesis level and had irregular structure evidenced in microscopic observations, disturbances in their auxin signal can be suspected. To sum up, low levels of active IAA, high levels of IBA precursor, and inactivated forms of auxins in haploid embryos, their irregular structure manifested in, e.g., "folded" scutellum, probably resulted from disorders in auxin synthesis, signaling, or transport during their morphogenesis. This conclusion is consistent with the necessity to use synthetic auxin in the oat DHs production protocol. It is worth noting that, the distinction of 'active' and 'inactivated' hormone forms is somehow arbitrary. Whiles Ostin et al.^45^ climes that IAA-Asp and IAA-Glu are irreversibly inactivated recent work of Tang et al.^46^ demonstrates the possibility of remobilization of thus conjugated IAA. Despite this suggestion, the role of auxins in the formation of haploid embryos definitely requires further research. 

In mature (dough stage) oat zygotic embryos the level of active cytokinins was low and IP precursor predominated. IP precursor was also a dominant form of cytokinin in oat haploid embryos, which were, however, richer in active cytokinins t-Z and DH-Z. This situation was similar to that in developing wheat embryos^21^ but different than in barley, where predominating t-Z and c-Z were accompanied by trace amounts of DH-Z^47^. Cytokinins are crucial in the embryo morphogenesis during intensive cell divisions, and later on at the maturation stage their content decreases^18^. Higher levels of cytokinins in haploid than in zygotic embryos suggested their earlier stage of development. This was consistent with microscopic observations of the embryos. As demonstrated by Skoog and Miller^48^ cytokinin to auxin ratio is important for plant organogenesis in vitro. This ratio diminishes during maize grain development^38^. In oat haploid embryos, the ratio of active cytokinins to active auxins was 0.6 and 0.4, while in the zygotic embryos it was 0.04 and 0.02 for cvs. ‘Krezus’ and ‘Akt’, respectively. This tenfold higher cytokinin to auxin ratio also indicated an earlier stage of development in the haploid embryos.

Oat embryos, similarly as *Cicer anatolicum*, *Cicer arietinum*, *Pisum sativum*, *Vicia faba*, or *Brassica napus*, contain gibberellins generated in two metabolic pathways (13-hydroxylation and non-13-hydroxylation pathway)^34,35,49^. In the zygotic embryos of cv. ‘Akt’ we detected only one precursor (GA9) and one form of active gibberellin (GA6). Contrary to that, cv. ‘Krezus’ contained all determined gibberellins except for GA7. This also suggested different rates of embryo maturation in these cultivars. There were no differences in the content of individual active gibberellins between haploid and zygotic embryos of the cv. ‘Krezus’. The high variability of data for the haploid embryos, may on the one hand indicate differences in stage of development of the embryos and on the other be ascribed to gibberellin metabolism that allows for their easy conversion from one form to another. This is in line with the reports by Hu et al.^50^, who observed dynamic changes in both the expression of genes related to the biosynthesis of active gibberellins (GA3ox and GA20ox) and their deactivation (GA2ox) between 1 to 17th DAP. Haploid embryos of cv. ‘Akt’ contained more GA9 precursor and all determined active gibberellins than the zygotic embryos, which suggested an earlier developmental stage of the former. Gibberellins are necessary at an early stage of embryogenesis, as they affect the formation of the embryo axis in *Brassica napus*^51^ and elongation of the dividing cells. Oat haploid embryos featured high concentrations of GA3. Similarly, maize kernels accumulated active gibberellins GA1 and GA3 during early embryogenesis, before ABA peak^52^. GAs and ABA maintained their antagonistic relationship of controlling seed germination (GAs) and dormancy (ABA).

The role of salicylic acid as a factor enhancing somatic embryogenesis in *Avena nuda* or *Astragalus adsurgens* is well known^53,54^. Likewise, abscisic, salicylic, and jasmonic acids added to a medium at an appropriate concentration may increase the efficiency of microsporogenesis in *Brassica napus*^55^. Although stress hormones are involved in the regulation of biological processes, including embryogenesis, their high concentrations may also indicate oxidative stress in the system. In the haploid embryos, we detected a few times higher levels of salicylic and jasmonic acids than in the zygotic ones. SA dampens auxin signaling by inhibiting the expression of auxin-associated genes, such as TIR1 receptor gene, with consequences for the stabilization of repressor protein AUX/IAA and reduced auxin signaling. JA acts synergistically with auxins^56^. High levels of salicylic acid (approx. 7000–13,000 pmol/g DW) in haploid embryos may negatively affect their auxin signaling and be related to the observed developmental disorders of haploid embryos.

In zygotic embryos, the level of abscisic acid rises and reaches its peak at the stage of embryo maturation when the seed enters its dormancy^57^. During embryogenesis of wheat, two ABA peaks were observed: a smaller one about 20 DAP and a larger one around 40 DAP^58,59^. It can be assumed that high ABA level (ca. 6000 pmol/g DW) in zygotic embryos of cv. ‘Krezus’ was related to their maturation and initiation of dormancy. The higher concentration of abscisic acid in haploid than zygotic embryos of cv. ‘Akt’ was not due to their maturation, as the haploid embryos were still at the stage of embryogenesis, but rather indicated strong stress, as ABA is not necessary for normal embryo formation at early embryogenesis^60^. This finding encouraged us to investigate oat embryos for possible oxidative stress. Indeed, a higher level of low molecular weight antioxidants and stress hormones in haploid than zygotic embryos suggests the presence of oxidative stress. To verify this hypothesis, other components of the embryo antioxidant system need to be examined.

## Conclusions

To the best of our knowledge, this is the first study comparing the anatomical structure and hormonal profile of haploid and zygotic oat embryos. Our experiments demonstrated that poor conversion of oat haploid embryos into plants was due to their immaturity. Twenty-one days-old embryos are at the early stages of development, which increases the risk of ‘embryo rescue’ failure. Irregular morphology and anatomy of haploid embryos and low content of endogenous auxins suggest disturbances in auxin signaling. High levels of stress hormones and non-enzymatic low-molecular-weight antioxidants may indicate the presence of oxidative stress in oat haploid embryos, but the role of oxidative stress in their embryogenesis requires further research. Our study can be helpful in improving the 'embryo rescue' methods and may contribute to a better understanding of the mechanisms controlling the development of haploid embryos.

## Supplementary Information

Supplementary Information.
