# Supplementary material for: Anatomical and hormonal factors determining the development of haploid and zygotic embryos of oat (Avena sativa L.)
Source: Sci Rep. 2022 Jan 11;12:548. doi: 10.1038/s41598-021-04522-y (PMC8752813; doi:10.1038/s41598-021-04522-y)
Supplement: Supplementary file 1 — Supplementary Information. [file 41598_2021_4522_MOESM1_ESM.docx]

Supplementary materials for:

Anatomical and hormonal factors determining the development of haploid and zygotic embryos of oat (*Avena sativa* L.)

Kinga Dziurka ^1^*, Michał Dziurka ^1^, Ewa Muszyńska-Sadłowska ^2^, Ilona Czyczyło-Mysza ^1^, Marzena Warchoł ^1^, Katarzyna Juzoń ^1^, Kamila Laskoś ^1^ and Edyta Skrzypek ^1^

1 Department of Biotechnology, The Franciszek Górski Institute of Plant Physiology, Polish Academy of Sciences, Niezapominajek 21, 30-239 Kraków, Poland

2 Department of Developmental Biology, The Franciszek Górski Institute of Plant Physiology, Polish Academy of Sciences, Niezapominajek 21, 30-239 Kraków, Poland

3 Department of Botany, Institute of Biology, Warsaw University of Life Sciences-SGGW, Nowoursynowska 159, Building 37, 02-776 Warsaw, Poland

*corresponding author e-mail: k.dziurka@ifr-pan.edu.pl, phone: +48 12 4251833

**Table S1**. Optimized mass spectrometry parameters for phytohormone quantitation. The following conditions were found optimal for the analysis: capillary voltage 4 kV, gas temperature 350 °C, gas flow 12 l/min and nebulizer pressure of 35 psi. The measurements were conducted by multiple reaction monitoring (MRM) in positive polarity. MassHunter software was used to control the LC-MS/MS system and for data analysis. For MRM parameters MassHunter Optimizer was used.

| Compound |  | Type of ion | Transition  (precursor/product ions) | Fragmentor voltage (V) | Collision energy (V) | MRM Start Time (min.) |
| --- | --- | --- | --- | --- | --- | --- |
| t-Z-7-G |  | [M+H]^+^ | 382.1/220.1 | 122 | 17 | 1 |
| t-Z |  | [M+H]+ | 220.2/136.3 | 85 | 9 |  |
| t-Z-O-G |  | [M+H]^+^ | 382.1/202.1 | 142 | 17 |  |
| c-Z-7-G |  | [M+H]^+^ | 382.1/220.1 | 122 | 17 |  |
| DHZ-N15 | ISTD | [M+H]+ | 226.2/152 | 124 | 18 |  |
| c-Z |  | [M+H]+ | 220.2/136.3 | 85 | 9 |  |
| c-Z-O-G |  | [M+H]^+^ | 382.1/202.1 | 142 | 17 |  |
| K-N15 | ISTD | [M+H]+ | 220.1/192.3 | 90 | 9 | 4.7 |
| K |  | [M+H]+ | 216.1/188.3 | 90 | 9 |  |
| GA8 |  | [M-H_2_O+H]^+^ | 319.3/257.2 | 102 | 9 |  |
| OxIAA |  | [M+H]^+^ | 192.2/146.1 | 54 | 9 |  |
| t-Z-R-D5 | ISTD | [M+H]^+^ | 357.3/225.2 | 116 | 17 | 5.9 |
| t-Z-R |  | [M+H]^+^ | 352.2/220.3 | 120 | 9 |  |
| DH-Z-R |  | [M+H]^+^ | 354,2/222,1 | 124 | 14 |  |
| c-Z-R |  | [M+H]^+^ | 352.2/220.3 | 120 | 9 |  |
| IP |  | [M+H]^+^ | 204.1/148.3 | 90 | 9 | 7.3 |
| IAA-Asp |  | [M+H]^+^ | 291.2/130.1 | 54 | 25 |  |
| KR |  | [M+H]^+^ | 204,1/148,3 | 90 | 9 | 9.1 |
| IAA-Glu |  | [M+H]^+^ | 305.2/130.1 | 58 | 29 |  |
| GA3-D2 | ISTD | [M-H2O+H]+ | 331.2/314.1 | 100 | 14 | 11 |
| GA3 |  | [M-H2O+H]+ | 329.3/311.3 | 100 | 14 |  |
| GA1-D2 | ISTD | [M-H_2_O+H]^+^ | 333.3/287.2 | 58 | 9 |  |
| GA1 |  | [M-H2O+H]+ | 331.3/285.3 | 100 | 14 |  |
| I3CA |  | [M+H]^+^ | 162.2/118.1 | 58 | 9 |  |
| IAA-D5 | ISTD | [M+H]+ | 181.1/135.1 | 38 | 14 |  |
| IAA |  | [M+H]+ | 176.1/130.3 | 51 | 9 |  |
| SA-D4 | ISTD | [M+H]+ | 143.2/125.2 | 80 | 14 | 11.3 |
| SA |  | [M+H]+ | 139.2/121.2 | 80 | 14 |  |
| ABA-glc |  | [M-H_2_O+H]^+^ | 409.2/247.1 | 104 | 14 | 12.0 |
| GA6-D2 | ISTD | [M-H_2_O+H]^+^ | 331.3/115.1 | 96 | 5 | 13.2 |
| GA6 |  | [M-H2O+H]+ | 329.3/283.3 | 104 | 14 |  |
| IPR |  | [M+H]^+^ | 336.2/204.1 | 124 | 14 |  |
| tt-ABA |  | [M-H2O+H]+ | 247.4/187.2 | 80 | 14 |  |
| ABA-D6 | ISTD | [M-H2O+H]+ | 253.4/191.3 | 80 | 14 | 17.5 |
| ABA |  | [M-H2O+H]+ | 247.4/187.2 | 80 | 14 |  |
| GA5-D2 | ISTD | [M-H_2_O+H]^+^ | 287.3/115.0 | 96 | 5 | 18.0 |
| GA5 |  | [M-H_2_O+H]^+^ | 285.1/115.0 | 96 | 5 |  |
| 4Cl-IAA |  | [M+H]^+^ | 210.4/164 | 80 | 14 | 18.8 |
| MeIAA-D5 | ISTD | [M+H]+ | 195/134 | 54 | 9 |  |
| MeIAA |  | [M+H]+ | 190/130 | 54 | 9 |  |
| JA-D5 | ISTD | [M+H]^+^ | 216.3/153.2 | 80 | 5 | 19.5 |
| JA |  | [M+H]^+^ | 211.3/151.2 | 80 | 14 |  |
| 5Cl-IAA |  | [M+H]^+^ | 210.4/164 | 80 | 14 |  |
| IBA |  | [M+H]^+^ | 204.1/186.4 | 69 | 9 |  |
| GA7 |  | [M-H_2_O+H]^+^ | 313.2/223.1 | 104 | 14 | 20.9 |
| GA4-D2 | ISTD | [M-H2O+H]^+^ | 317.3/271.2 | 88 | 9 |  |
| GA4 |  | [M-H2O+H]^+^ | 315.3/269.3 | 100 | 14 |  |
| GA9 |  | [M-H2O+H]^+^ | 271.3/225.2 | 136 | 13 | 23.1 |
| dinor-12-oxo-OPDA-D5 | ISTD | [M+H]^+^ | 270.3/252.2 | 84 | 5 | 23.72 |
| 12-oxo-PDA |  | [M+H]^+^ | 293.3/275.2 | 68 | 9 | 25.54 |


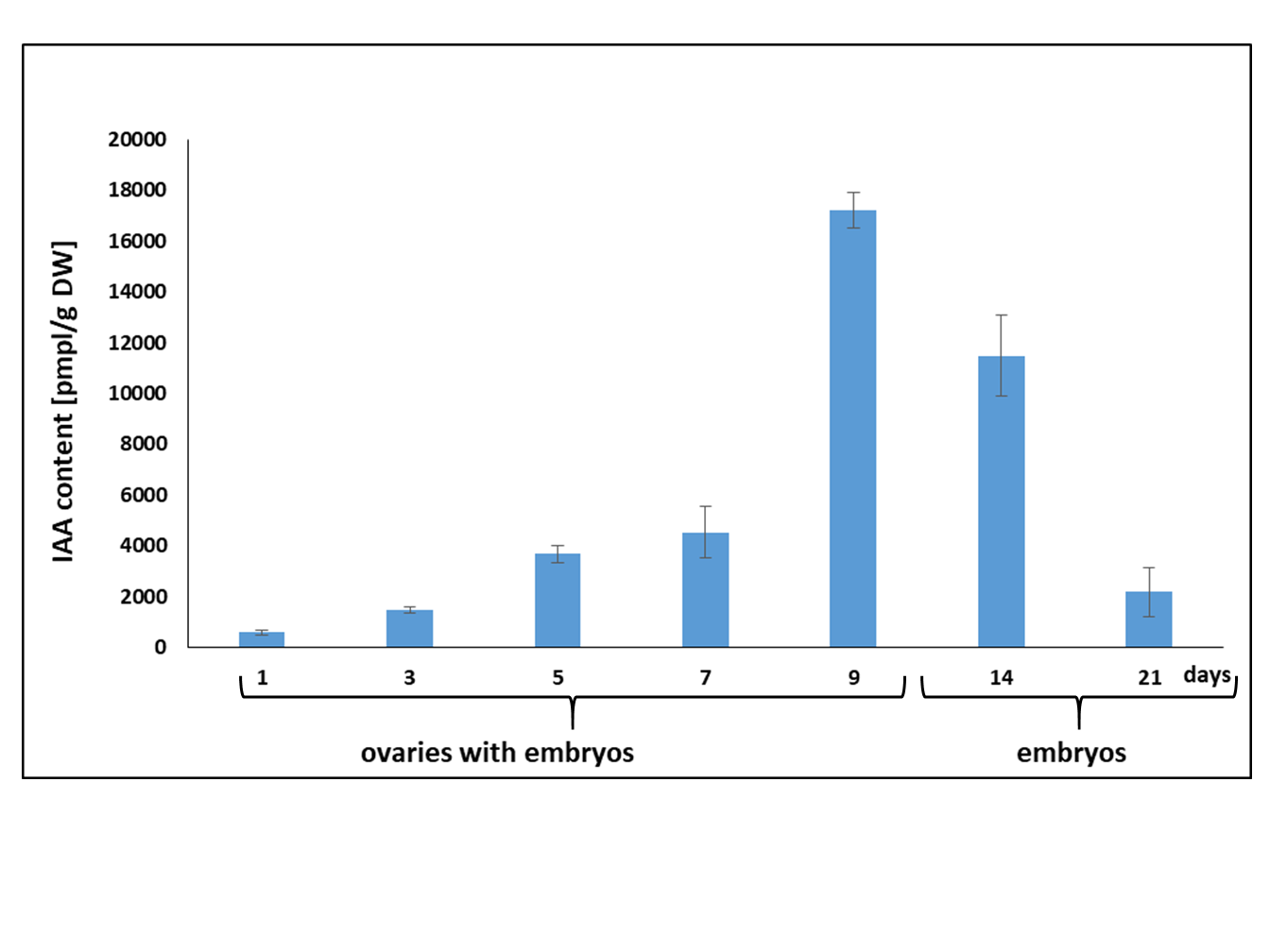


**Figure S1.** IAA content during oat cv. ‘Krezus’ kernel development. Values are the mean of triplicates ± SE.
